# Bleomycin-Induced Flagellate Dermatitis

**DOI:** 10.4274/tjh.galenos.2019.2018.0317

**Published:** 2019-05-03

**Authors:** Esra Turan Erkek, Ceren Nur Karaali, Güven Yılmaz, Emine Gültürk

**Affiliations:** 1 Lütfi Kırdar Training and Research Hospital, Clinic of Hematology, İstanbul, Turkey; 2 Bahçeşehir University Faculty of Medicine, İstanbul, Turkey

**Keywords:** Bleomycin, Hodgkin lymphoma, Flagellate dermatitis

## To the Editor,

Bleomycin is a cytostatic, antineoplastic antibiotic that is used in both of the first-line treatments of Hodgkin lymphoma: ABVD (doxorubicin, bleomycin, vinblastine, dacarbazine) and BEACOPP (doxorubicin, bleomycin, vincristine, cyclophosphamide, etoposide, prednisone, procarbazine). The bleomycin hydrolase enzyme metabolizes bleomycin. This enzyme is not found in the skin or lung tissues; therefore, bleomycin accumulates in those areas and causes side effects [1]. The dermatologic side effects of bleomycin may vary from onycholysis, pruritus, and scleroderma-like skin changes to Stevens-Johnson syndrome. Flagellate dermatitis, resulting after bleomycin therapy, was originally described by Moulin et al. [2] in 1970 as “bleomycin-induced linear hyperpigmentation” [3]. Although the term “flagellate dermatitis” was described for bleomycin-induced dermatitis, other causes of this symptom have been defined over time (Table 1) [4]. The characteristic symptoms are pruritic linear hyperpigmentations, arranged in a flagellate pattern and developing, in particular, on the trunk. Even though the exact mechanism is not clear, minor skin traumas are thought to be responsible since they increase blood flow to the affected area and cause drug accumulation [1].

**Figure 1:**
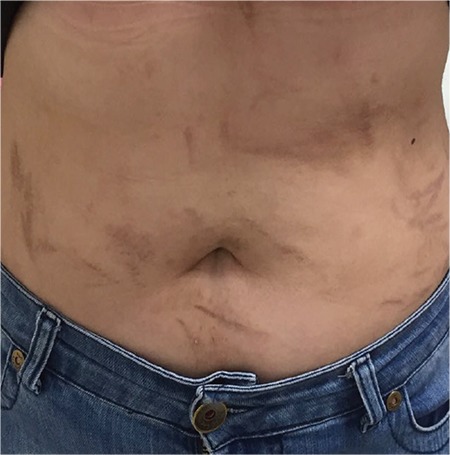
Flagellate dermatitis on trunk.

**Figure 2:**
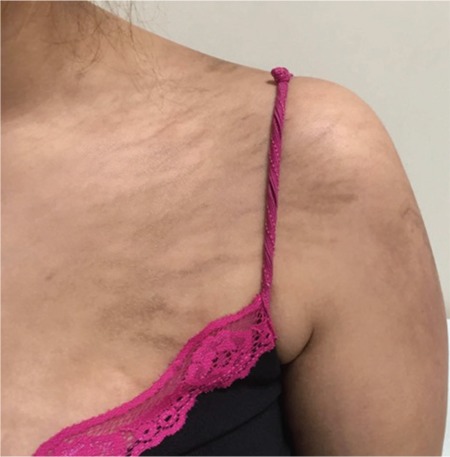
Flagellate dermatitis on extremity.

We present a 24-year-old female patient who was diagnosed in August 2016 with stage IIA Hodgkin lymphoma (right cervical, submandibular, and bilateral palatine tonsil involvement was observed in positron emission tomography/computed tomography). A BEACOPP chemotherapy regimen was chosen for first-line therapy. After the second cycle of BEACOPP, the patient developed generalized and intense pruritus along with the appearance of papules and plaques on her back, shoulders, and trunk, with a remarkable whip-like mark formation (Figures 1 and 2), which evolved into hyperpigmentation. There was no evidence of mucosal or systemic involvement. Contrary to expectations, there was no evidence of dermatographia. Flagellate dermatitis was diagnosed by the clinical features. The patient did not have a history of dermatomyositis, Still’s disease, hypereosinophilic syndrome, or shiitake mushroom intake. The BEACOPP regimen was interrupted after three cycles of chemotherapy were completed. The skin lesions started to resolve two weeks after the bleomycin-inducing therapy was suspended.

Bleomycin-induced flagellate dermatitis is a dose-dependent reaction that usually occurs with total doses above 100 U [5,6]. In contrast with these results, some patients develop skin symptoms after low doses. The incidence of developing flagellate dermatitis and consequent hyperpigmentation after receiving bleomycin treatment is reported between 8% and 22% [7]. The lesions usually diminish 3-4 months after the interruption of the bleomycin treatment. Other than the suspension of the bleomycin treatment, no effective treatment has been reported for bleomycin-induced ﬂagellate dermatitis. In the literature, there are some cases that report the use of topical or systemic corticosteroid treatments, as well as oral antihistamine treatments. However, it is stated that those treatments provide only symptomatic relief. The cessation of bleomycin is necessary to prevent further relapse [8]. We found it worthwhile to present our case since the development of this condition is rarely seen after a low dosage, the lesions disappear shortly after the suspension of the medication, and flagellate dermatitis is not observed with the other medications that our patient was receiving. Clinicians must be aware of this uncommon complication and act immediately to interrupt the causative agent.

## Figures and Tables

**Table 1:**
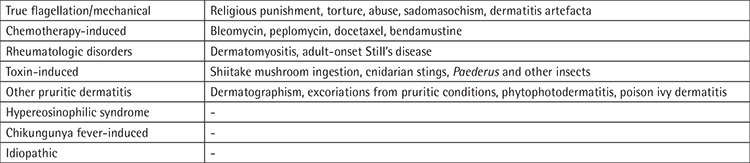
Causes of flagellate dermatitis.
